# Nonadiabatic dynamics of cobalt tricarbonyl nitrosyl for ligand dissociation induced by electronic excitation

**DOI:** 10.1038/s41598-021-88243-2

**Published:** 2021-04-26

**Authors:** Yeonghun Lee, Grigory Kolesov, Xiaolong Yao, Efthimios Kaxiras, Kyeongjae Cho

**Affiliations:** 1 Department of Materials Science and Engineering, University of Texas at Dallas, Richardson, TX 75080 USA; 2 John A. Paulson School of Engineering and Applied Sciences, Harvard University, Cambridge, MA 02138 USA; 3 Department of Physics, Harvard University, Cambridge, MA 02138 USA

**Keywords:** Atomistic models, Electronic structure, Chemical physics, Synthesis and processing

## Abstract

We utilize real-time time-dependent density functional theory and Ehrenfest dynamics scheme to investigate excited-state nonadiabatic dynamics of ligand dissociation of cobalt tricarbonyl nitrosyl, Co(CO)_3_NO, which is a precursor used for cobalt growth in advanced technologies, where the precursor’s reaction is enhanced by electronic excitation. Based on the first-principles calculations, we demonstrate two dissociation pathways of the NO ligand on the precursor. Detailed electronic structures are further analyzed to provide an insight into dynamics following the electronic excitations. This study sheds light on computational demonstration and underlying mechanism of the electronic-excitation-induced dissociation, especially in molecules with complex chemical bonds such as the Co(CO)_3_NO.

## Introduction

The scaling limit for the current complementary metal–oxide semiconductor (CMOS) technology necessitates a three-dimensional integration with vertically stacked electronic components^1^. This requires low thermal budget processes in order to prevent the degradation of devices and interconnects underneath the top layer undergoing thermal processes. Electron-enhanced atomic layer deposition (EE-ALD) can be a solution to create reactive dangling bonds at low temperatures by using an energetic electron beam^2–4^. In addition to the EE-ALD, the energetic electrons can be exploited for focused electron beam induced deposition (FEBID), which is a useful technique for nanopatterning on solid surfaces^5^. Cobalt has been paid a lot of attention as a promising interconnect material. The continuous increase in the density of transistors keeps reducing the metal interconnect width. However, further scaling of current copper and tungsten interconnects is challenging due to their requirement of a thick diffusion barrier, which brings about a narrow conductive cross-sectional area. This limitation becomes rapidly severe as scale decreases. To overcome the issue, barrierless cobalt can be a promising interconnect material to replace the copper and tungsten^6^. Also, cobalt enables us to pattern FEBID nanostructures for catalytic and ferromagnetic applications^7,8^. Here, a widely used organometallic cobalt precursor is cobalt tricarbonyl nitrosyl, Co(CO)_3_NO^4,9,10^. The high metal content of this compound is associated with low incorporation of impurities, such as C, N, and O, resulting in high conductivity.

The electron-induced dissociation of ligands on the Co(CO)3NO has been investigated experimentally in gas phase^11–14^ or in liquid helium nanodroplets^15^. A reaction of the gas phase Co(CO)3NO can mimic that on an inert oxide surface. This corresponds to the initial stage of a cobalt film deposition, which is typically slow and requires to be accelerated. The electron-induced dissociation occurs once energy is transferred from the energetic electrons to the target via inelastic electron scattering processes. Photodissociation also involves the pertinent excitation-driven phenomenon, where excitation is introduced in internal degrees of freedom of the molecule. The photodissociation of ligands on the Co(CO)_3_NO has also been studied in previous experimental works^16–20^. While experimental progress based on such excitation-induced dissociation of the Co(CO)_3_NO has been actively reported in terms of relatively long-time processes by taking into account intramolecular vibrational energy redistribution, there have been very few theoretical studies providing microscopic insights on the process. Specifically, a direct ligand dissociation pathway has not yet been demonstrated, and the first-principles dynamics simulation can play a role in elucidating the ultrafast phenomenon.

This work elucidates the atomic and electronic processes that constitute the excitation-driven dissociation of ligands on Co(CO)_3_NO using the first-principles calculation based on the density functional theory (DFT)^21,22^. The molecular dynamics (MD) within the Born–Oppenheimer approximation^23^, however, cannot describe such nonadiabatic dynamics along with electronic excitation. To deal with nonadiabatic processes in the coupled electron–ion systems, we employ the real-time time-dependent density functional theory (TDDFT)^24,25^ and Ehrenfest dynamics scheme^26–29^. In the present study, we locate direct dissociation channels, thus providing an insight into the excitation-driven unimolecular dissociation of the Co(CO)_3_NO.

## Methods

### TDDFT-MD simulation

To explore excited-state nonadiabatic dynamics, we carry out ab initio molecular dynamics study employing the TDDFT-MD based on Ehrenfest dynamics, which has been implemented in a computational package, TDAP-2.0 (Time-evolving Deterministic Atom Propagator)^28–32^ based on the SIESTA^33^ package. The exchange–correlation (XC) energy functional is given by the Perdew-Burke-Ernzerhof (PBE) functional^34^ in the generalized gradient approximation (GGA), and the adiabatic approximation^25^ is employed for the XC functional in the TDDFT. The GGA functional is a reasonable choice not only to reproduce experimental measurements^35^ but also to maintain consistency with TDDFT works based on pure DFT methods. The norm-conserving pseudopotentials are constructed with Troullier–Martins scheme^36^. A localized, atom-centered, numerical atomic-orbital basis set is employed. We use the double-ζ polarized (DZP) basis set configuration and perform spin-polarized calculations. The real-space mesh cutoff is 120 Ry, and we use 13 Å × 13 Å × 13 Å simulation cell. Convergence tolerance of the density matrix is set to 10^–4^. The time step for integration is 0.5 ħ/Ry (1 Hartree a.u.)^28,32,37,38^, which is reliable as we did not observe any divergence in physical observables. We have performed a convergence test with different mesh cutoffs, resulting in the 120 Ry of the mesh cutoff exhibits enough convergence in the total energy. We have also tested single-ζ polarized (SZP) basis set, where the SZP shows the same key features as the DZP in time-evolving Co-X bond lengths with different excitations. Therefore, the larger DZP basis set would be a safe choice. We note that a dipole correction should be employed to eliminate the spurious electric field induced by the periodic boundary condition. The artificial electric field accelerates ionic motion, often leading to erroneous dissociation.

In the Ehrenfest dynamics, ions are approximated by classical particles evolving on the mean-field average of adiabatic potential energy surfaces (PESs); thus, detailed balance^39^, spontaneous phonon emission^40,41^, and zero-point motion^30,40^ are missing. As to the Co(CO)_3_NO, the validity of the Ehrenfest approximation will be discussed in the following section. Furthermore, the adiabatic XC functional depends only on the instantaneous electronic density, neglecting memory effects (time-non-locality), which involve the initial-state dependence and history dependence^25^. Despite these limitations, the Ehrenfest dynamics and the adiabatic XC functional have been utilized widely in most practical applications^28–32,37,38,42–45^. For instance, reaction pathways of photodissociation of water on the rutile TiO_2_(110) surface and photo-oxidation of CH_3_O on the same surface have been illustrated with the same scheme^29,31^.

### Protocol of ligand dissociation modeling

**Figure 1:**
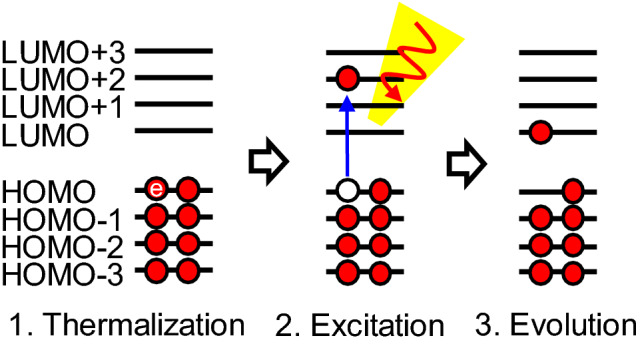
Sequence of TDDFT-MD modeling of reaction induced by electronic excitation.

Figure 1 illustrates the TDDFT-MD modeling of ligand dissociation driven by electronic excitation. First, at the ground state, geometry is optimized until the maximum force is below the tolerance value of 0.04 eV/Å. Then, equilibration at room temperature is performed with the ground-state Born–Oppenheimer molecular dynamics. After that, an excited state is created using the Δ self-consistent field (ΔSCF) method^46,47^, which simulates an electronic excitation induced by an inelastic electron scattering or a photoexcitation. For instance, an electron is promoted from the highest occupied molecular orbital (HOMO) to the third-lowest unoccupied molecular orbital (LUMO + 2), which is followed by orbital relaxation within the ΔSCF method. Finally, we let the excited system evolve in time, where coupled electron–ion dynamics is described by the TDDFT-MD. When extracting static microscopic quantities, the geometry optimized at the ground state is used.

## Results and discussion

### Electronic structure calculations

**Figure 2:**
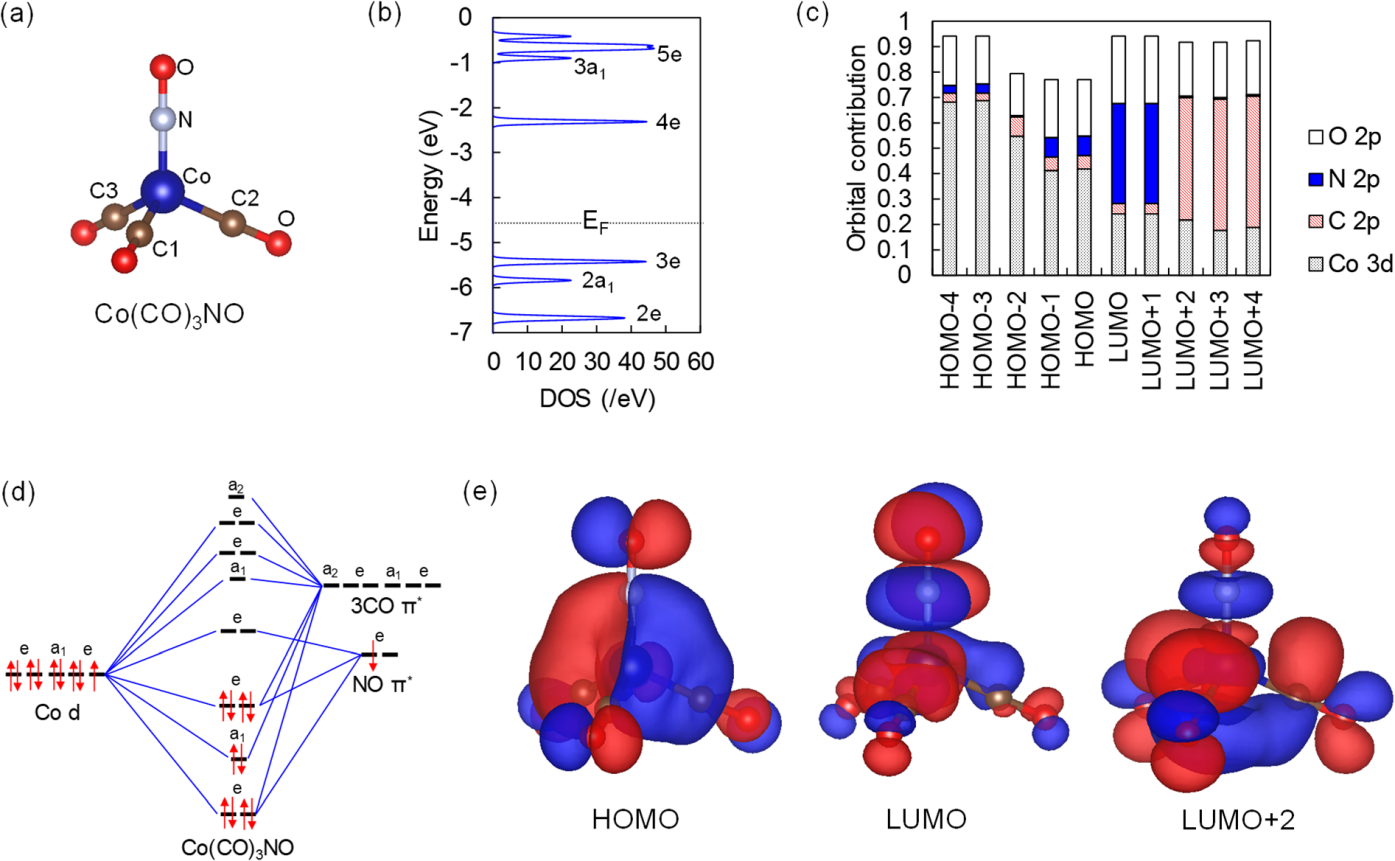
Molecular geometry and electronic structures in the ground state. (**a**) Atomistic structure of Co(CO)_3_NO. (**b**) DOS around the Fermi level, ${E}_{F}$. (**c**) KS eigenstates decomposed into orbital contributions. (**d**) Molecular orbitals for Co(CO)_3_NO with the C_3v_ point group. (**e**) Wavefunction of HOMO, LUMO, and LUMO + 2. Two different colors—blue and red—are assigned to positive and negative isosurfaces.

In the ground state, the organometallic precursor Co(CO)_3_NO has a distorted tetrahedral geometry belonging to the C_3v_ point group with the Co–N–O bond being linear in the axial position (Fig. 2a)^48,49^. First, we analyze the electronic ground state (singlet). Figure 2b shows the density of states (DOS) around the Fermi level, and Fig. 2c shows contributions of Co 3*d*, C 2*p*, N 2*p*, and O 2*p* orbitals to those energy levels. The orbitals around the Fermi level are described by the metal *d* and ligand π backbond. More specifically, empty π^*^ orbitals of CO and NO contribute to bonds with Co *d* orbital. The molecular orbital diagram for Ni(CO)_4_, which has the same number of electrons with the Co(CO)_3_NO but has a higher symmetry, helps us to understand where the molecular orbitals of the Co(CO)_3_NO originate from. The diagram shown in Figure S1 describes how the π backbond forms in the Ni(CO)_4_^50^. Compared with the Ni(CO)_4_, the replacement of a CO group by a NO group changes the point group from T_d_ into C_3v_ in the Co(CO)_3_NO and lifts degeneracies as shown in Fig. 2d. ^35^. The HOMO 3*e* orbitals exhibit the π backbond between Co and ligands. Looking at unoccupied states, the LUMO and LUMO + 1 are doubly degenerate 4*e* orbitals, the LUMO + 2 is a nondegenerate 3*a*_1_ orbital, and the LUMO + 3 and LUMO + 4 are doubly degenerate 5*e* orbitals. The bonding characteristics turn out to be clearer when we analyze the real-space distribution of those wavefunctions. As illustrated in Fig. 2e, the LUMO and LUMO + 1 are an antibonding state formed by Co *d* and the NO π^*^-antibonding state, and the LUMO + 2, LUMO + 3, and LUMO + 4 are likewise an antibonding state formed by Co *d* and the CO π^*^-antibonding state. We exploit these distinguishing LUMO and LUMO + 2 states to simulate excited-state dynamics for ligand dissociation, where an electron is promoted from the HOMO to the LUMO (3*e* to 4*e*) or from the HOMO to the LUMO + 2 (3*e* to 3*a*_1_). The excitation operation is done within the same spin channel in such a way that a spin-up (spin-down) electron is promoted to a spin-up (spin-down) empty state. Thus, a sum of singlet and triplet states describes the excitation as usual in a single-determinant approach^51–53^.

### Standard process driven by single-electron excitation

**Figure 3:**
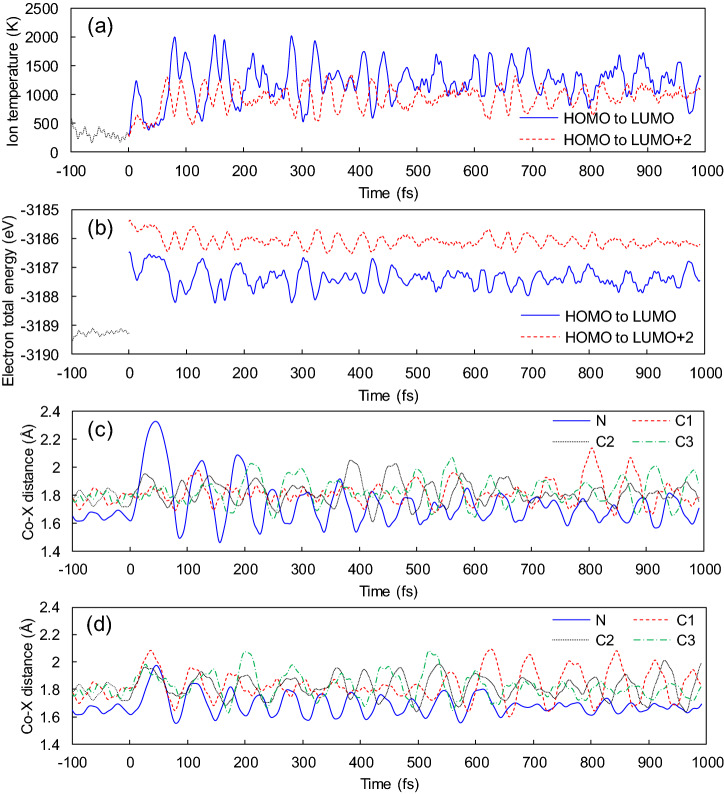
Excited-state dynamics. (**a**) Ion temperature and (**b**) electron total energy as functions of time, where the electron total energy includes ion-ion potential energy. First, systems are equilibrated at 300 K from − 100 to 0 fs; next, HOMO-to-LUMO and HOMO-to-LUMO + 2 excitations are induced at 0 fs; then, the excited states evolve in time from 0 to 1000 fs. Time evolution of Co-X distances induced by (**c**) HOMO-to-LUMO and (**d**) HOMO-to-LUMO + 2 excitations (X = N, C1, C2, C3).

Figure 3 shows the results of coupled electron–ion dynamics driven by the HOMO-to-LUMO and HOMO-to-LUMO + 2 excitations. Here, ion temperature corresponds to ion kinetic energy, and the electron total energy includes the ion-ion potential energy. During the initial equilibration (from − 100 to 0 fs), the molecule reaches room temperature (Fig. 3a). Then, the excitations are forced at 0 fs, and electron total energies increase abruptly (Fig. 3b). During subsequent dynamics, ion temperature increases up to around 1000 K for both the excitations; at the same time, electron total energy decreases, where the summation of them remains constant. Once the excitations are introduced, the electronic state changes; thus, forces are spontaneously exerted on atoms (Table S1). These exerted forces drive ion vibration followed by electron dynamics. Keeping track of interatomic distances between Co and adjacent atoms, the vibrations are damped, and the initially localized vibrational energy is redistributed eventually (Fig. 3c,d). This indicates that rapid dissociation is unlikely to happen with the given conditions.

### Systems with simple σ bonds

We further investigated excited-state dynamics initiated by various excitations in different combinations of unoccupied and occupied molecular orbitals (Table S2) and driven by ionization and electron attachment (Figure S2). The results revealed that dissociation is unlikely to occur with a single electron process without an additional driving force. The Co–N or Co–C bond is a triple bond consisting of a σ bond and a pair of π backbonds. This complex bonding nature between the metal and ligands prevents dissociation after promoting a single electron. On the other hand, simple σ bonds in H_2_, H_2_O, and CH_4_ facilitate hydrogen dissociation with the HOMO-to-LUMO one-electron excitation (Figure S3a-c). As in Co(CO)_3_NO, immediate dissociation is unlikely to happen in OCS because four electrons are responsible for each bond by forming σ and π bonds, simultaneously (Figure S3d).

### Analysis of time evolution of Kohn–Sham orbital energy levels

**Figure 4:**
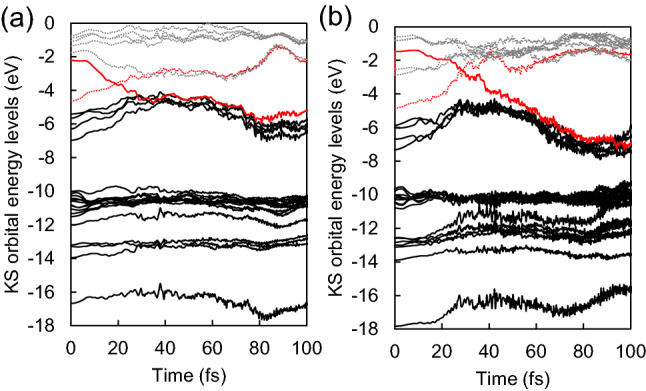
Time evolution of spin-up KS orbital energy levels driven by (**a**) HOMO-to-LUMO excitation and (**b**) HOMO-to-LUMO + 2 excitation. Solid lines and dotted lines are associated with occupied states and empty states, respectively. The red lines indicate time-evolving energy levels of the initial HOMO and (**a**) LUMO [(**b**) LUMO + 2] states. Internal conversion to the electronic ground state happens within 50 fs in both cases.

For further investigation of electron dynamics, we examine the time evolution of Kohn–Sham (KS) orbital energy levels, which are expectation values of the KS Hamiltonian with respect to time-evolving KS orbitals. Although it is known that the absolute value of KS orbital energy levels hardly possesses physical meaning, relative values provide useful information, such as a transition from an electronically excited state to the ground state, as shown in Fig. 4. Here, the applicability of Kasha’s rule^54^ and the validity of the Ehrenfest dynamics for the system can be discussed with the transition of the electronic states. Kasha’s rule is applicable when energy differences between S_*n*_ (*n*th-excited state) and S_1_ are small, and the energy difference between S_0_ (ground state) and S_1_ is large so that an electronic state can stay at the S_1_ for a long time. Figure 4a shows that the lowest excited state, S_1_, only lasts for a short time less than 50 fs. Within that time, internal conversion (IC) to the electronic ground state is accompanied by ion dynamics. Since the lifetime of the S_1_ is too short for the S_1_ to manage molecular dissociation, Kasha’s rule would not be applicable to the dissociation dynamics of the Co(CO)_3_NO. Guo et al.^55^ have also been reported such ultrafast IC dynamics in energetic dimethylnitramine, where the lifetime of S_1_ is 50 ± 16 fs. When it comes to the HOMO-to-LUMO + 2 excitation (Fig. 4b), the state is rapidly converted into the S_1_, which seems to be consistent with Kasha’s rule; however, the short lifetime of S_1_ implies breaking Kasha’s rule as well.

In the TDDFT-MD, forces exerted on ions are derived using the Ehrenfest dynamics scheme, which is valid in the absence of the mean-field average of PESs. To avoid mixing of PESs, the energy difference between PESs is required to be large enough, which is observed during most of the time of excited-state dynamics of Co(CO)_3_NO. Figure 4 shows that the occupied orbital energy level originally higher goes down while the empty orbital energy level goes up, where those two levels get close for a short time less than 10 fs. Therefore, except for this short period, the motion of ions is primarily described in a single PES; eventually, after 50 fs, the electronic ground state governs the system. The validation we made for the Ehrenfest dynamics has also been discussed in excited carrier dynamics of carbon nanotubes and in photoisomerization of azobenzene^38,43–45^. As demonstrated above, the Ehrenfest dynamics allows us to perform on-the-fly simulations of the particular system without considering trajectory-based methods, such as fewest-switches surface hopping^56^ although the subtle quantum nature of ion dynamics remains elusive. Regarding the zero-point motion^30,40^, which is neglected in the classical picture, will be insignificant because the ion temperature is 300 K or higher.

### Potential energy curves and vibration-excitation processs

**Figure 5:**
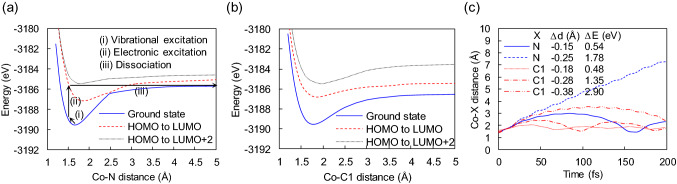
Ligand dissociation induced by a combination of vibrational and electronic excitations. (**a**) Adiabatic potential energy curves as functions of Co–N distance. Arrows with descriptions (i–iii) are associated with a possible pathway of Co–N dissociation induced by HOMO-to-LUMO excitation. (**b**) Adiabatic potential energy curves as functions of Co–C1 distance. (**c**) Time evolution of Co-X distance during coupled electron–ion dynamics initiated by HOMO-to-LUMO excitation. Before the excitation, Co-X distance is initially adjusted by $\Delta d$, and the molecule gains energy by $\Delta E$, which is regarded as vibrational excitation at the electronic ground state.

Sobell et al*.* have demonstrated experimentally that the electron beam facilitates ligand dissociation, which cannot be explained by a typical thermal activation due to substrate heating but can be explained by electronic excitation^4^. Nevertheless, our results so far do not show any instantaneous ligand dissociation. For further investigation, we computed potential energy curves as functions of Co–N and Co-C1 distances (Fig. 5a,b). Based on the potential energy curves, a possible instantaneous dissociation pathway of the NO ligand on the precursor is depicted in Fig. 5a: (i) the Co–N bond is vibrationally excited, (ii) once the bond gains sufficient vibrational energy, electronic excitation is introduced along with the Franck–Condon principle, and (iii) the system evolves in time and overcomes the dissociation limit. It is worth noting that from the small gap at the crossing point in the adiabatic representation shown in Fig. 5a, a crossing of potential energy curves of the ground state and the HOMO-to-LUMO excited state is expected to be observed in the diabatic representation. Thus, a transition from the first excited state to the ground state can readily occur during the time evolution, which reduces the energy required for the dissociation; however, the barrier is still too high to see dissociation at room temperature without initial vibrational excitation. Comparing bond energies of Co–N and Co-C1 in the ground state shown in Fig. 5a,b, the bond energy of Co–N is greater than that of Co–C1, indicating that the Co–N bond seems to be more difficult to break. Interestingly, electronic excitation can make Co–N break more easily than Co–C1. Figure 5c shows time evolutions of interatomic distances starting with vibrational excitations followed by electronic excitation. The result implies that Co–N bond breaking is more frequent as the thermal energy required for Co–N bond breaking is smaller than that of Co–C1 bond breaking. Through intramolecular vibrational energy redistribution, some electronic excitations can be converted to vibrational energy, which can cause molecular heating sufficient to realize the proposed dissociation even at room temperature; however, the barrier is still too high to see dissociation at room temperature without initial vibrational excitation. This combined process illustrates the stimulated ligand dissociation, which cannot be explained solely by either electronic excitation or thermal activation.

### Two-electron process

Another scenario of the electronic-excitation-driven dissociation can involve a two-electron process. In our previous work, we have studied the electron-Co(CO)_3_NO scattering process for the EE-ALD using the TDDFT^57,58^. It has turned out that the maximum energy transferred from the incident electron wave packet to the molecule can reach approximately 10 eV when the kinetic energy of the incident electron is around 170 eV, which is within the typical energy range used for the EE-ALD^4^. Since the HOMO–LUMO gap is 3.1 eV, the excitation energy of the 10 eV is sufficient to promote two electrons from the HOMO to the LUMO. The two-electron excitation is less likely to happen right after the electron-molecule collision. Having transferred sufficient energy to the molecule, however, the additional excited electron can be generated as in the multiple exciton generation^59,60^. Also, two-electron excitation can be induced by the Knotek-Feibelman mechanism, in which secondary Auger electrons play a leading role in further excitation of the surface molecule^61^. Consecutive scatterings by two electrons or two photons can also be considered as a source of the two-electron excitation. The detailed mechanism and quantitative analysis for the two-electron excitation processes mentioned above could further legitimate the proposed mechanism, albeit beyond the scope of this study.

**Figure 6:**
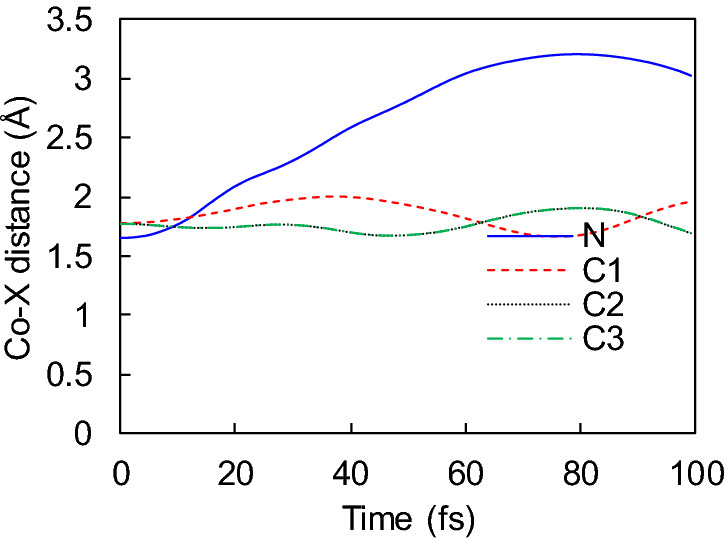
Co–N bond breaking driven by a two-electron excitation from the HOMO to the LUMO. This excitation can be achieved during electron-Co(CO)_3_NO scattering^57,58^.

The employed real-time adiabatic TDDFT can include double excitation with a superposition state, whereas the linear-response adiabatic TDDFT is incapable of dealing with multielectron excited states^62,63^. As a result of TDDFT-MD simulation starting with the two-electron HOMO-to-LUMO excitation, the Co–N bond breaks instantaneously (Fig. 6), which brings about rapid dissociation before the substate or molecule heating. Although the Co–N bond is not fully broken in Fig. 6, the broad amplitude of the band length indicates that a small amount of additional thermal energy or interaction with other molecules can readily cause dissociation. Also, the Co–C1 bond length exhibits a different time evolution from Co–C2 and Co–C3 because of the Co–C1 bonding character different from others, as shown in Fig. 2e, where the N–Co–C1, N–Co–C2, and N–Co–C3 bond angles are 114.1°, 115.6°, and 115.6°, respectively. Interestingly, the adiabatic PEC shown in Figure S4 does not explain the instantaneous bond breaking of Co–N. Accordingly, nonadiabatic coupling can play a crucial role in the dissociation process induced by the two-electron excitation displayed in Fig. 6. However, in contrast to the constrained DFT^64^, our typical DFT has a limitation in describing charge or spin localized state when we construct an adiabatic potential energy curve. Therefore, the charge or spin localized state can also play a role in the instantaneous dissociation. Although we could not identify the detailed mechanism because of the limitation of our approach, it remains valid that the Co–N bond breaking can be induced by the two-electron excitation.

## Conclusion

In conclusion, using the Ehrenfest-TDDFT method, we investigated the microscopic details of Co(CO)_3_NO dissociation driven by electronic excitation and demonstrated two direct dissociation pathways of the NO ligand on the Co(CO)_3_NO. According to the first dissociation pathway, molecular vibration plays a vital role in the electronic-excitation-driven dissociation. Whereas either single-electron excitation or simple vibrational heating rarely gives rise to ligand dissociation, we found a direct dissociation pathway of the NO ligand on the Co(CO)_3_NO by introducing subsequent electronic excitation after vibrational excitation. Another instantaneous dissociation pathway was demonstrated along with a two-electron excitation, which can be induced by energy transferred from an incident electron to the molecule via the electron-molecule scattering. In those dissociation pathways, the CO ligand is less likely to dissociate from the Co(CO)_3_NO than the NO ligand although the Co–N has larger bond energy than Co–C in the ground state. However, further experimental and theoretical efforts are necessary to confirm the proposed pathways. In particular, an in-depth investigation of temperature-dependent growth rates in tandem with electronic excitations would allow one to differentiate the effects of vibrational and electronic excitations on ligand dissociation. Also, sophisticated simulation approaches in determining the two-electron excitation process could shed additional light on the proposed mechanism.

## Supplementary Information

Supplementary Information.
