# Supplementary material for: Nonadiabatic dynamics of cobalt tricarbonyl nitrosyl for ligand dissociation induced by electronic excitation
Source: Sci Rep. 2021 Apr 26;11:8997. doi: 10.1038/s41598-021-88243-2 (PMC8076320; doi:10.1038/s41598-021-88243-2)
Supplement: Supplementary file 1 — Supplementary Information. [file 41598_2021_88243_MOESM1_ESM.pdf]

Supplementary information:

"Nonadiabatic dynamics of cobalt tricarbonyl nitrosyl for ligand dissociation induced by electronic excitation"

Yeonghun Lee,[1] Grigory Kolesov,[2] Xiaolong Yao,[1] Efthimios Kaxiras,[2,3] and Kyeongjae Cho[1,*]

[1]Department of Materials Science and Engineering, University of Texas at Dallas, Richardson, TX 75080, USA

[2]John A. Paulson School of Engineering and Applied Sciences, Harvard University, Cambridge, MA 02138, USA

[3]Department of Physics, Harvard University, Cambridge, MA 02138, USA

Corresponding author

[*]Email: kjcho@utdallas.edu

Table S1. Interatomic forces exerted between Co and X atoms, F_{CoX}. Here, $\boldsymbol{F}_X$ and $\boldsymbol{F}_{Co}$ are vectors of force exerted on X and Co atoms, respectively, and θ is the angle between the two vectors. Once introducing electronic excitation in the ground-state geometry, we measure the interatomic force.

	$F_{CoX} = \lvert\boldsymbol{F}_X\rvert - \lvert\boldsymbol{F}_{Co}\rvert \cos(\theta)$ (eV/Å)		
X	Ground state	HOMO to LUMO	HOMO to LUMO+2
N	0.03	7.25	3.32
C1	0.02	1.06	-0.18
C2	0.02	2.79	1.87
C3	0.02	2.80	1.87

Table S2. Taking into account various excited states within the same spin state, we have checked whether rapid dissociation occurs within 200 fs. Blanks mean that ΔSCF does not converge for the particular excitation. Excitation is introduced within the geometry optimized at the ground state. These results indicate that dissociation hardly happens without additional processes.

From	To	Dissociation
HOMO	LUMO+7	
HOMO	LUMO+6	No
HOMO	LUMO+5	
HOMO	LUMO+4	No
HOMO	LUMO+3	
HOMO	LUMO+2	No
HOMO	LUMO+1	
HOMO	LUMO	No
HOMO-1	LUMO	
HOMO-2	LUMO	
HOMO-3	LUMO	No
HOMO-4	LUMO	
HOMO-5	LUMO	
HOMO-6	LUMO+2	No
HOMO-6	LUMO	No
HOMO-7	LUMO	
HOMO-8	LUMO	No
HOMO-9	LUMO	No

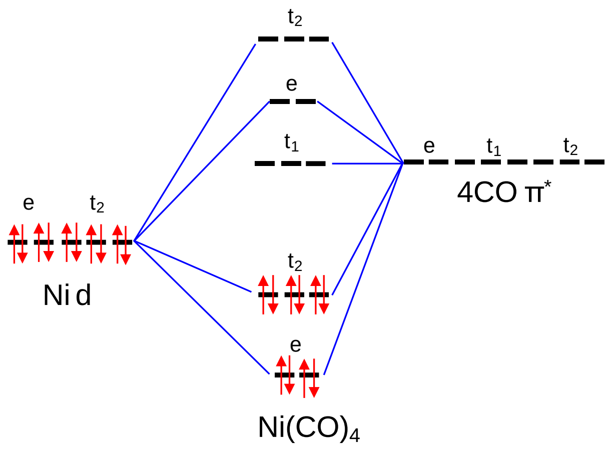

Figure S1. Molecular orbitals for $Ni(CO)_4$ with the T_d point group.

(a) q = +1

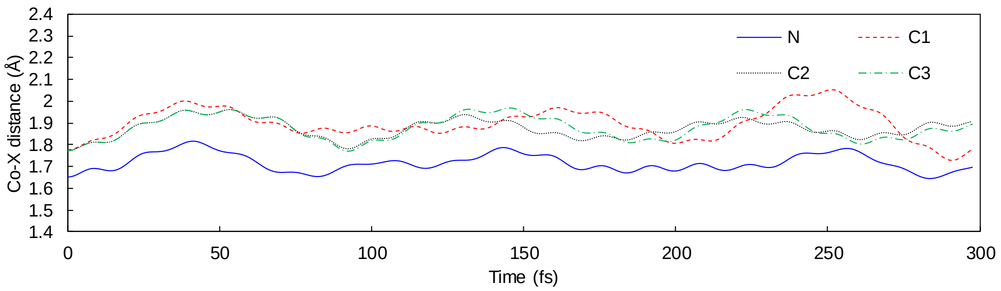

(b) q = -1

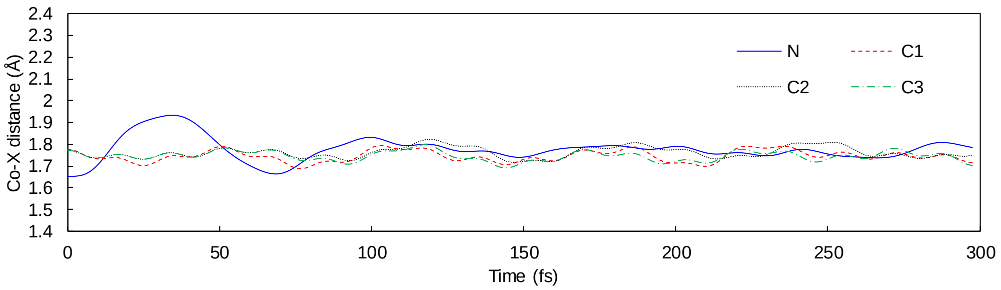

Figure S2. Time evolutions of Co-X distance during coupled electron-ion dynamics initiated by (a) ionization (q = +1) and (b) electron attachment (q = −1). Having optimized geometry at the ground state, we change the electron number and perform electronic optimization using ΔSCF.

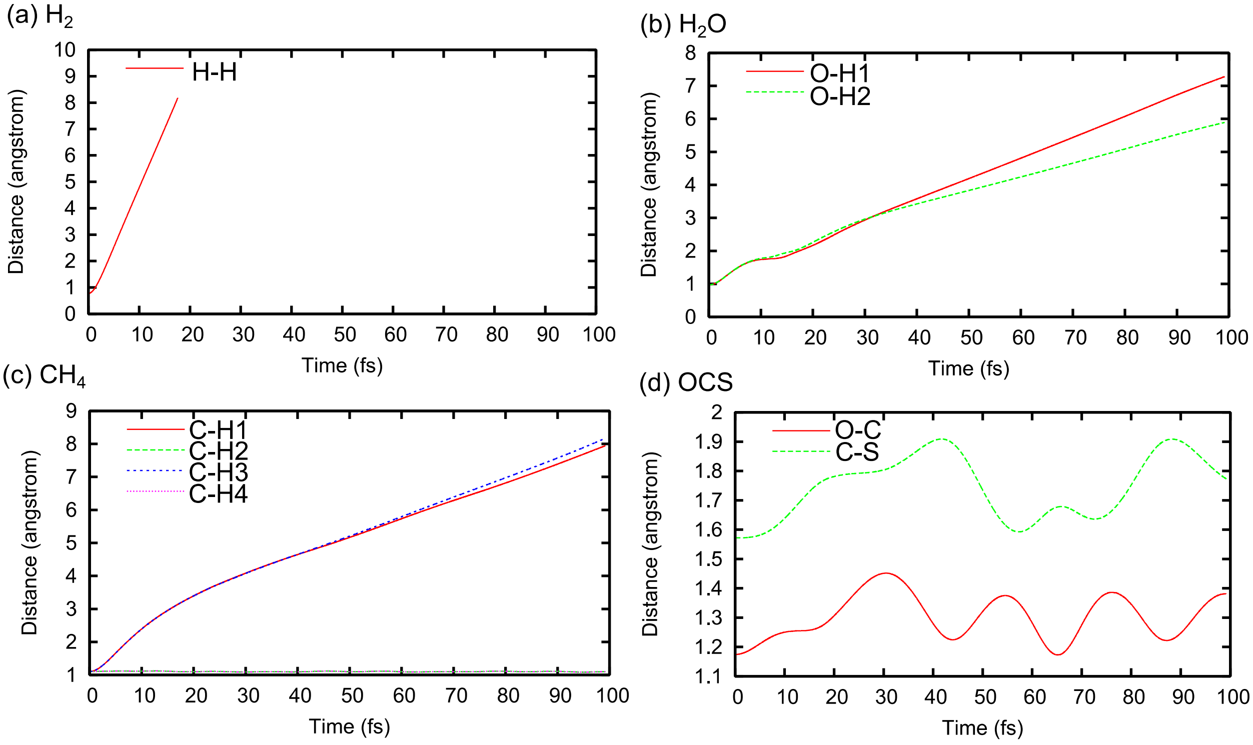

Figure S3. Interatomic distances in (a) H_2, (b) H_2O, (c) CH_4, and (d) OCS during nonadiabatic dynamics driven by HOMO-to-LUMO one-electron excitation. Having optimized geometry at the ground state, we introduce the excitation using ΔSCF and let the system evolve using TDDFT-MD. Simple σ bonds in H_2, H_2O, and CH_4 facilitate hydrogen dissociation with the given excitation. On the other hand, dissociation is unlikely to happen in OCS with the one-electron excitation because four electrons are responsible for each bond of the molecule by forming σ and π bonds at the same time.

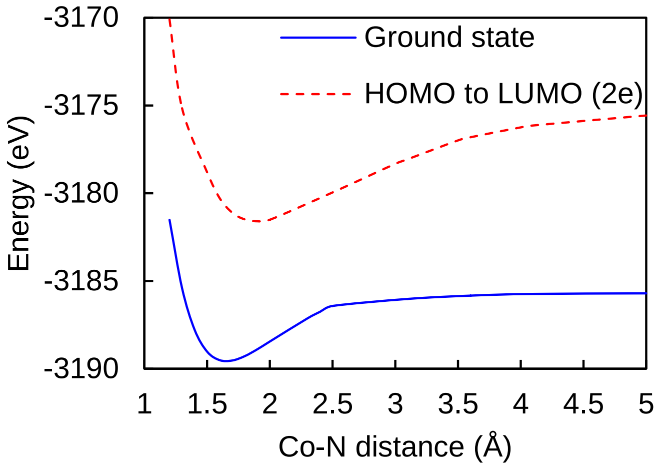

Figure S4. Adiabatic potential energy curves for two-electron HOMO to LUMO excitation.as functions of Co-N distance.
